# Screening of Lactic Acid Bacteria and RSM-Based Optimization for Enhancing γ-Aminobutyric Acid (GABA) Accumulation in Orange Juice

**DOI:** 10.3390/foods15010071

**Published:** 2025-12-25

**Authors:** Shufeng Yin, Yiyao Wang, RuiXue Zhao, Ning Zhao, Hao Liu, Yining Tang, Ningbo Qin, Yiwei Dai, Xinping Lin

**Affiliations:** State Key Laboratory of Marine Food Processing & Safety Control, National Engineering Research Center of Seafood, School of Food Science and Technology, Dalian Polytechnic University, Dalian 116034, China; yinshufeng1220@163.com (S.Y.); wangyiyao0506@163.com (Y.W.); zhaorx04@outlook.com (R.Z.); ningzhao0221@163.com (N.Z.); 18763666325@163.com (H.L.); 18904084306@163.com (Y.T.); qinningbo6902765@163.com (N.Q.)

**Keywords:** γ-aminobutyric acid (GABA), orange juice, *Lacticaseibacillus paracasei*, *Lacticaseibacillus rhamnosus*, single-factor experiment, response surface methodology (RSM), functional beverage

## Abstract

Inoculated fermentation can enhance the flavor, nutrition, and functionality of juice. The lactic acid bacteria (LAB) are commonly used as starter cultures. This study screened LAB for orange juice fermentation and optimized fermentation factors using response surface methodology (RSM) to improve GABA content in orange juice. A total of 52 LAB strains were screened, and *Lacticaseibacillus paracasei* ZY (*Lcb. paracasei* ZY) and *Lacticaseibacillus rhamnosus* SN12 (*Lcb. rhamnosus* SN12) presented higher GABA yields and adaptability to substrates. The optimized fermentation factors for GABA enhancement in orange juice were as follows: initial pH of 5.5, fermentation temperature of 37 °C, soluble solids content of 12.0 °Bx, inoculum ratio of *Lcb. paracasei* ZY to *Lcb. rhamnosus* SN12 as 1:1, inoculum size of 6 Log CFU/mL, and fermentation time of 96 h. Under these optimized conditions, the GABA content reached 0.89 g/L, representing a 39.06% increase compared to uninoculated orange juice. This indicates that RSM-based optimization is conducive to increasing GABA content in orange juice and provides a scientific basis for the development of GABA-enriched functional fermented juices.

## 1. Introduction

γ-aminobutyric acid (GABA) is a non-protein amino acid that is widely present in plants and animals. As a major inhibitory neurotransmitter in the central nervous system, GABA has multiple physiological functions, including improving sleep [1], alleviating anxiety [2], lowering blood pressure [3], and regulating blood sugar [4]. Compared with chemical synthesis and plant extraction methods, microbial fermentation is safer and more sustainable for GABA production [5]. Lactic acid bacteria (LAB), as generally recognized as safe (GRAS) probiotics, can irreversibly decarboxylate glutamic acid or monosodium glutamate (MSG) to convert GABA through glutamic acid decarboxylase (GAD). In recent years, they have received increasing attention in the food industry [6]. The reported species of GABA-producing LAB include *Levilactobacillus brevis* (*L. brevis*) [7], *Lactiplantibacillus plantarum* (*Lpb. plantarum*) [8], *Lacticaseibacillus rhamnosus* (*Lcb. rhamnosus*) [9], *Lacticaseibacillus paracasei* (*Lcb. paracasei*) [10], and *Limosilactobacillus fermentum* (*Lim. fermentum*) [11].

Oranges are rich in nutrients, containing carbohydrates, vitamin C, and polyphenols [12]. Their high juice content makes them highly attractive to consumers. At present, the main processed products of orange juice are concentrated juice and freshly squeezed juice. However, traditional processing methods are difficult to meet consumers’ growing demands for nutritional value. Fermentation, as an effective means to enhance flavor, nutritional value, and bioactive substances, has been widely applied in the production of juice. Due to their safety and probiotic properties, LAB are often used in the preparation of non-alcoholic fermented drinks. Wu et al. [13] reported that fermenting citrus juice with *Lpb. plantarum* A72 could significantly increase the contents of total phenols, flavonoids, and antioxidant activity. Quan et al. [14] evaluated the effect of six strains of LAB on orange juice fermentation and found that the orange juice fermented by *Lcb. paracasei* had the best flavor and overall sensory acceptance. Huang et al. [15] confirmed that fermentation with *Lactiplantibacillus paraplantarum* M23 could significantly enhance the antioxidant and antibacterial properties of orange juice. However, current research on fermented orange juice mainly focuses on its antioxidant activity, while discussions on other functional properties remain relatively limited.

GABA content found in natural foods tends to be low and may fail to meet biological requirements. While the direct addition of GABA to food products is possible as a food additive, its stability and safety cannot be guaranteed. GABA-producing LAB are effective in developing GABA-enriched fermented foods. Wang et al. [16] reported that, after litchi juice was fermented by *Lpb. plantarum* HU-C2W for 40 h, the GABA content increased by approximately 40 mg/100 mL. Zheng et al. [17] found that fermenting *Moringa oleifera leaves* with *Lpb. plantarum* LK-1 could increase the content of GABA, polyphenols, and organic acids. Kanklai et al. [18] demonstrated that, when fermenting mulberry juice with *L. brevis* F064A, the final GABA content reached 3.31 mg/mL. Jin et al. [19] reported that fermenting litchi juice supplemented with 0.5% MSG using *L. brevis* LBG-29 increased the GABA content to 3.07 g/L. In contrast, Zhou et al. [20] achieved only 0.22 g/L of GABA in persimmon juice through co-fermentation with *Lpb. plantarum* C17 and *L. brevis* Lp-B at a 1:1 ratio for 48 h at 30 °C. GABA production in fermented juices depends not only on the substrate but also on the interplay of key process variables such as temperature and pH. Thus, rational optimization of fermentation conditions is essential for enhancing GABA yield. The traditional single-factor optimization is difficult to accurately reflect the mutual influence among these factors. In contrast, the response surface methodology (RSM) can quantitatively analyze the interactions among the factors and establish a more reliable and efficient fermentation model. Wang et al. [21] optimized the fermentation conditions of soymilk using RSM, and the results showed that, by using *Lpb. plantarum* Lp3 and *Streptococcus thermophilus* in a ratio of 2:1 as the starter, adding 0.4% MSG at 35 °C for 10 h, the highest GABA content (0.55 mg/mL) could be obtained. It is significantly superior to a single strain or other co-fermentation combinations. Shan et al. [8] optimized the process of fermenting yogurt with *Lpb. plantarum* NDC75017 using RSM and found that an MSG concentration of 80 mM, pyridoxal phosphate at 18 μM, and a fermentation temperature of 36 °C produced a maximum GABA content of 314.56 mg/100 g.

In this study, two high GABA-producing LAB strains were identified through the Berthelot colorimetric method and high-performance liquid chromatography (HPLC) screening. Their fermentation performance and probiotic properties were systematically evaluated, and the suitability of orange juice as a fermentation substrate for LAB growth was assessed. A single-factor experiment combined with RSM was employed to optimize initial pH, fermentation temperature, soluble solids content, inoculum ratio, inoculum size, and fermentation time, aiming to produce GABA-enriched orange juice. These findings provide a theoretical foundation and process guidance for the development of functional fermented juices.

## 2. Materials and Methods

### 2.1. Starter Preparation

In the initial stage of the experiment, 52 strains of LAB were isolated from fermented foods to construct a GABA-producing screening library (Table A1). The strains were streaked onto MRS agar (Haibo, Qingdao, China) and then incubated at 37 °C for 48 h. Subsequently, single colonies were incubated in MRS broth (Haibo, Qingdao, China) at 37 °C for 16–20 h to reach a cell density of 6–8 log CFU/mL. Bacterial cells were collected by centrifugation (6000× *g*, 5 min, 4 °C) and resuspended in sterile saline. The suspension was inoculated into MRS broth containing 10 g/L MSG (99% purity; Meihua, Tongliao, Inner Mongolia, China) to achieve an initial cell density of approximately 6 log CFU/mL, followed by incubation at 37 °C for 48 h. The culture medium without LAB served as the control. All experimental equipment was sterilized by autoclaving at 121 °C for 15 min, and all procedures were performed under aseptic conditions.

### 2.2. GABA-Producing LAB Screening

#### 2.2.1. Rapid Screening of GABA by Berthelot Colorimetric Method

The Berthelot colorimetric method was slightly revised based on the method reported by Zhang et al. [22]. LAB fermentation cultures prepared in Section 2.1 were subjected to centrifugation at 6000× *g* for 10 min at 4 °C, and 0.5 mL of the obtained supernatant was aliquoted. Subsequently, 1 mL of 6.0% (*w*/*v*) phenol (Macklin, Shanghai, China), 0.4 mL of 7.0% (*w*/*v*) sodium hypochlorite (Sangon Biotech, Shanghai, China), and 0.2 mL of 0.2 mol/L borate buffer (pH 9.0; Damao, Tianjin, China) were added and thoroughly mixed. The reaction mixtures were incubated in a boiling water bath for 7 min and then immediately cooled in an ice bath for 10 min. Subsequently, 2 mL of 60% (*v*/*v*) ethanol (Fuyu, Tianjin, China) was added, and the absorbance was measured at 630 nm. The top 15 LAB strains with the highest GABA production were further quantified by HPLC.

#### 2.2.2. Quantitative Screening of GABA by HPLC

The GABA content in the fermented orange juice and the top 15 LAB fermentation cultures (Section 2.1) were determined using an Essentia LC-16 HPLC system (Shimadzu Ltd., Tokyo, Japan) according to Wu et al. [13]. After centrifugation at 6000× *g* for 10 min at 4 °C, an aliquot of 200 μL from the resulting supernatant was subjected to derivatization with ortho-phthaldialdehyde (OPA; Aladdin, Shanghai, China) for 5 min, followed by filtration through a 0.22 μm membrane filter. Subsequently, 10 μL of the filtrate was injected into a HPLC system for analysis. Chromatographic separation was carried out on an Agilent Extend-C18 column (4.6 mm × 250 mm, 5 μm) with a mobile phase composed of acetonitrile (phase A) and 50 mM sodium acetate solution (phase B) under an isocratic elution of 35:65 (*v*/*v*). The column temperature was kept at 30 °C, the mobile phase flow rate was set to 0.5 mL/min, and detection was performed at 334 nm.

### 2.3. Evaluation of LAB Fermentation Performance and Probiotic Properties

High GABA-producing LAB were cultured in MRS broth, centrifuged, and resuspended as described in Section 2.1. LAB were inoculated into 96-well plates containing 200 μL of MRS broth to an initial OD_600_ of 0.2, and growth at 37 °C was monitored every 2 h using a Bioscreen C automated growth curve analyzer (Oy Growth Curves Ab Ltd., Turku, Finland). Acid tolerance (pH 3.0, 2 h) and bile salt tolerance (0.03% *w*/*v*, 3 h) were assessed following Meena et al. [23]. Simulated Gastric Fluid (SGF) and Simulated Intestinal Fluid (SIF) tolerance were evaluated as described by Huang et al. [24], with results expressed as survival rates (%).

### 2.4. Evaluation of Substrate Adaptation

High GABA-producing LAB were cultured in MRS broth, and centrifuged as described in Section 2.1. After being washed twice with sterile saline, the LAB were resuspended in saline and inoculated into orange juice to achieve an initial cell density of approximately 6 Log CFU/mL. The orange juice was fermented at 37 °C for 48 h. Viable cells were counted on MRS agar using the standard plate count method and expressed as log CFU/mL.

### 2.5. Preparation of Fermented Orange Juice

Oranges were purchased from the Qianhe Market (Dalian, Liaoning, China), and fermented orange juice was prepared following the method of Wu et al. [13]. After peeling and deseeding, the fruits were homogenized with an equal volume of deionized water and filtered through three layers of cheesecloth to obtain clarified juice. Before fermentation, 8 g/L MSG was added. The juice was pasteurized at 97 °C for 3 min using a vertical pressure steam sterilizer (DSX-30L-I, Shanghai SHENAN Medical Device Co., Ltd., Shanghai, China). Bacterial cells were prepared for inoculation as described in Section 2.4. When cooled to room temperature (25 °C), LAB were inoculated into the orange juice. Orange juice fermentation was conducted under static conditions, with the orange juice without LAB set as the control group. Fermentation conditions were adjusted according to the experimental design, and collected samples were stored at −80 °C for later analysis within 2 weeks.

### 2.6. Design of Single-Factor Experiment

The influence of initial pH, fermentation temperature, soluble solids content, inoculum ratio, inoculum size, and fermentation time on GABA production by GABA-producing LAB in orange juice was evaluated through single-factor experiments (Table A2). The aim was to determine the optimal range of each factor in RSM. The initial pH of orange juice was adjusted with sodium bicarbonate and measured using a pH meter (FE28 Standard, Mettler Toledo, Greifensee, Switzerland). The content of soluble solids content was adjusted with glucose and measured using a handheld refractometer (LYT-380, ATAGO, Tokyo, Japan), and the results were expressed as °Bx.

### 2.7. Design of RSM

#### 2.7.1. Plackett–Burman Design

Based on the findings from the single-factor experiments, the Plackett–Burman design was utilized to determine significant factors for GABA yields from fermented orange juice [25]. In this design, the GABA content was taken as the response value (Y), while the initial pH (A), fermentation temperature (B), soluble solids content (C), inoculum ratio (D), inoculum size (E), and fermentation time (F) were selected as the Plackett–Burman design factors (Table A3).

#### 2.7.2. Box–Behnken Design

Based on the significant factors identified by the Plackett–Burman design, a Box–Behnken design (Table A5) was employed to investigate the interactive effects among each factor [26]. Non-significant factors were fixed at their optimal levels established by single-factor experiments.

### 2.8. Model Validation

Triplicate experiments were performed under the optimized fermentation conditions to verify the model derived from RSM.

### 2.9. Statistical Analysis

Experiments were carried out in three separate replicates, with outcomes reported as the mean ± standard deviation (mean ± SD). Statistical evaluation was performed using SPSS (version 24.0; IBM Corp., Armonk, NY, USA). Data visualization was accomplished with Origin (version 2021b; OriginLab, Northampton, MA, USA), while RSM modeling and analysis were conducted through Design-Expert software (version 13.0; Stat-Ease, Minneapolis, MN, USA). The experimental runs of the Plackett–Burman design (Table A4) and Box–Behnken design (Table A6) were randomly generated using Design-Expert software (version 13.0; Stat-Ease, Inc., Minneapolis, MN, USA).

## 3. Results and Discussion

### 3.1. Analysis of GABA-Producing LAB

**Figure 1:**
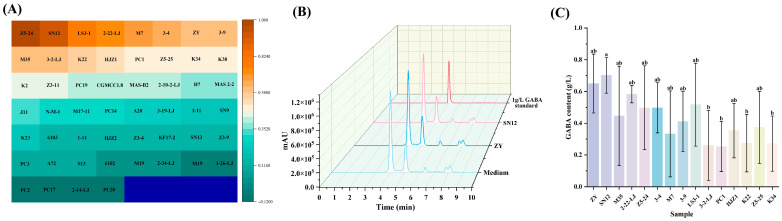
GABA determination via the Berthelot colorimetric method (**A**), HPLC chromatogram of GABA (**B**), and GABA quantification by HPLC (**C**). Different letters indicate significant differences among different treatments (*p* < 0.05).

As shown in Figure 1A, 52 LAB isolates were rapidly screened via the Berthelot colorimetric method, among which forty-three strains exhibited GABA-producing ability. The top five LAB strains were identified as *Lcb. paracasei* ZY (1.00 g/L), *Lcb. rhamnosus* SN12 (0.82 g/L), *Lpb. plantarum* M35 (0.75 g/L), *Lpb. plantarum* 2-22-LJ (0.71 g/L), and *Lpb. plantarum* Z5-24 (0.68 g/L). To further validate these results, the top 15 GABA-producing LAB strains identified by the Berthelot colorimetric method were recultured and quantified using HPLC. Figure 1B shows the chromatograms of the medium, the LAB strains (*Lcb. paracasei* ZY and *Lcb. rhamnosus* SN12), and the 1 g/L GABA standard, while the HPLC results are shown in Figure 1C. HPLC analysis confirmed that the highest GABA producers were *Lcb. paracasei* ZY (0.65 g/L), *Lcb. rhamnosus* SN12 (0.70 g/L), *Lpb. plantarum* 2-22-LJ (0.58 g/L), *Lpb. plantarum* LS3-1 (0.52 g/L), and *Lpb. plantarum* Z5-24 (0.50 g/L). We found that GABA content measured by the Berthelot colorimetric method was generally higher than that obtained by HPLC, consistent with the findings of Le et al. [27]. This is because the Berthelot colorimetric method lacks specificity, and other amino-containing substances in the sample react with it, leading to an overestimation of GABA content. Considering the GABA production ability of the strains and their stability during fermentation, *Lcb. paracasei* ZY and *Lcb. rhamnosus* SN12 were ultimately selected as the subjects for subsequent research.

### 3.2. Analysis of Fermentation Performers and Probiotic Properties

**Figure 2:**
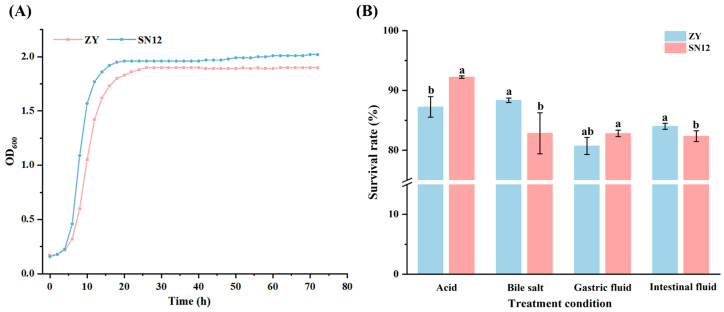
Fermentation performance (**A**) and probiotic properties (**B**) of *Lcb. paracasei* ZY and *Lcb. rhamnosus* SN12. Different letters indicate significant differences among different treatments (*p* < 0.05).

Both *Lcb. paracasei* ZY and *Lcb. rhamnosus* SN12 have shown classic ‘S’ curves for growth (Figure 2A). In the first 6 h of incubation, growth was slow for both strains, followed by log phase growth. *Lcb. paracasei* ZY and *Lcb. rhamnosus* SN12 entered the stationary phase at 20 and 24 h of culture, respectively. At the end of 72 h culture, neither strain showed a significant decrease in absorbance, thus making them good fermentation performers.

Figure 2B shows the tolerance of the strains to acids, bile salts, SGF, and SIF. At an acidic pH 3.0, both strains were able to sustain a survival rate of more than 87.00%, while *Lcb. rhamnosus* SN12 showed 92.23% survival at that pH, thereby displaying high-level tolerance to highly acidic environments. Since 0.3% bile salt concentration is equivalent to its approximate concentration found in the human small intestine [28], this concentration was tested. In MRS both containing 0.3% bile salt, both strains maintained survival rates over 82.00%, demonstrating strong resistance to bile salts. Both strains exhibited survival rates exceeding 80.00% in SGF and SIF, which was higher than that of *L. brevis* DSM 32386 and DSM 20054 [7], displaying strong resistance to digestive enzymes. Both strains showed excellent probiotic properties and hold potential as starter cultures for food fermentation.

### 3.3. LAB Adaptability Evaluation in Orange Juice

**Figure A1:**
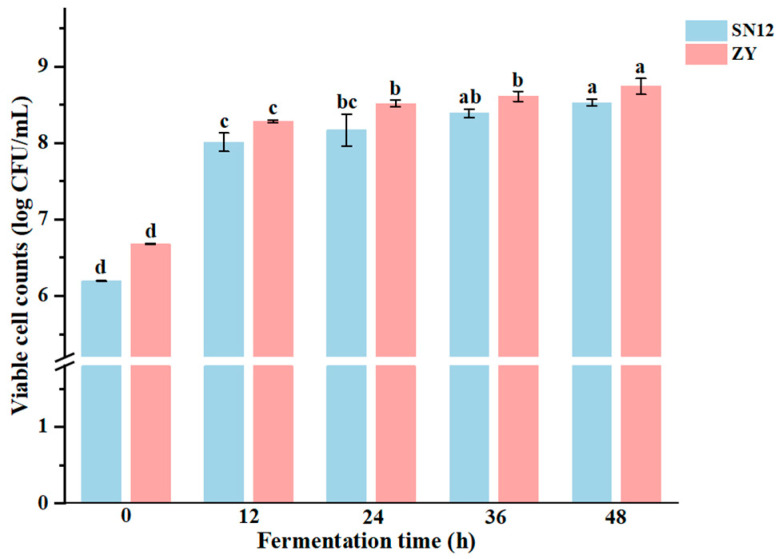
Viable cell counts of *Lcb. paracasei* ZY and *Lcb. rhamnosus* SN12 in orange juice during fermentation.

Adaptability is also very important for applying strains to orange juice. Based on Figure A1, it is shown that *Lcb. paracasei* ZY and *Lcb. rhamnosus* SN12 have an upward trend in orange juice. At the initial growth stage of fermentation, the viable cell counts for both LAB strains were 6.68 and 6.19 log CFU/mL, respectively. In the initial 12 h of fermentation, both LAB strains grew very quickly and then entered a period of relatively slow growth. After 48 h, the viable cell counts of *Lcb. paracasei* ZY and *Lcb. rhamnosus* SN12 reached approximately 8.74 and 8.53 log CFU/mL, respectively, and tended to stabilize. These results are consistent with the findings of Jin et al. [19], who reported a significant increase in the population of *L. brevis* within the first 16 h of litchi fermentation (*p* < 0.05), followed by a stable phase. Therefore, the two LAB strains can maintain excellent adaptability in orange juice.

### 3.4. Analysis of Single-Factor Experiment

There have been studies exploring GABA synthesis by LAB influenced by fermentation processes [6]. In this study, we selected initial pH, fermentation temperature, soluble solids content, inoculum ratio, inoculum size, and fermentation time as optimization factors.

#### 3.4.1. pH

**Figure 3:**
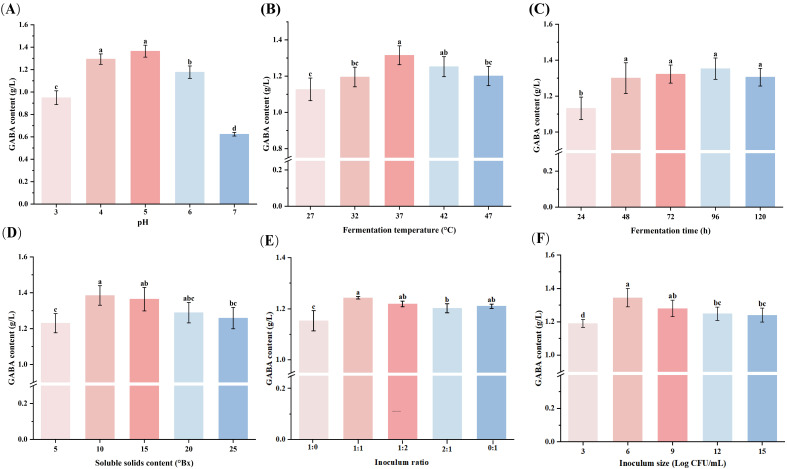
Effects of single factors on GABA production in fermented orange juice, including initial pH (**A**), fermentation temperature (**B**), soluble solids content (**C**), inoculum ratio (**D**), inoculum size (**E**), and fermentation time (**F**). Different letters indicate significant difference among different treatments (*p* < 0.05).

pH is very significant for GAD activity for GABA synthesis. Previous studies have shown that most LAB exhibit enhanced metabolic activity under mildly acidic conditions [29]. In our experiments (Figure 3A), GABA production increased as pH increased to reach its peak at 1.36 g/L at pH 5.0 and started to reduce gradually. This optimal pH likely reflects the maximum GAD activity at pH 5.0, facilitating the efficient conversion of Glutamic acid and MSG to GABA. Similarly, Komatsuzaki et al. [10] also found that *Lcb. paracasei* NFRI 7415 produced 210 mM GABA at an initial pH of 5.0, which was markedly higher than production at pH 4.0 or 6.0 (*p* < 0.05).

#### 3.4.2. Fermentation Temperature

Temperature is another important factor to consider during fermentation. As illustrated in Figure 3B, GABA content in orange juice rose with increasing temperature up to 37 °C, but began to decrease when the temperature exceeded this point. Based on single-factor optimization experiments focusing on temperature, 37 °C is likely the optimal fermentation temperature, given that GAD enzyme activity is significantly enhanced at this temperature. This result is also identical to Komatsuzaki et al. [10], who observed that *Lcb. paracasei* NFRI 7415 produced the highest content of GABA (302 mmol/L) at 37 °C, with a notable drop at 43 °C. Rayavarapu et al. [30] also applied RSM to optimize GABA production in soymilk fermented with *Lim. fermentum* and found that the maximum GABA content (4.2 g/L) was also achieved at 37 °C.

#### 3.4.3. Soluble Solids Content

The soluble solids content had a significant effect on the accumulation of GABA in fermented orange juice. As shown in Figure 3C, the GABA content reached its maximum when the soluble solids content was 10.0 °Bx. This result was attributed to the balance between carbon source supply and osmotic regulation. An adequate carbon source not only provides the necessary substrates and energy for cell growth, but also maintains the permeability and metabolic activity of the cell membrane [31].

#### 3.4.4. Inoculum Ratio

Figure 3D shows the effect of the inoculation ratio on the accumulation of GABA in fermented orange juice. When the inoculum ratio of *Lcb. paracasei* ZY to *Lcb. rhamnosus* SN12 was 1:1, the GABA content was the highest (1.36 g/L). This is because at this ratio, the combination of the two LAB strains’ metabolism resulted in the effective utilization of substrates for higher GABA concentration.

#### 3.4.5. Inoculum Size

Figure 3E shows the influence of inoculum size on GABA content in orange juice. It was found that 6 Log CFU/mL inoculum size resulted in a higher GABA content than any other inoculum size. This suggests that a moderate initial cell density helps the strain establish dominance more quickly, boosting metabolic activity and ultimately increasing GABA synthesis. A similar trend was observed by Rayavarapu et al. [30], who studied soymilk fermentation with *Lim. fermentum* and found that a 5.8 Log CFU/mL inoculum size led to the highest GABA content among the tested levels.

#### 3.4.6. Fermentation Time

LAB start to produce GABA during the logarithmic growth phase and continue through to the stationary growth phase [32]. Figure 3F presents the effect of fermentation time on GABA content in orange juice. GABA content showed significant increments from 0 to 96 h (*p* < 0.05), while, from 96 to 120 h, the increment was very slight (*p* > 0.05). This shows that LAB start to produce GABA between 24 and 96 h. After 96 h, the accumulation of GABA stopped due to the consumption of substrates (such as glutamic acid) and the production of lactic acid by LAB, which led to a decrease in pH value.

Based on single-factor experiments, the most suitable factor levels for GABA accumulation in orange juice are the following: initial pH of 5.0, fermentation temperature of 37 °C, soluble solids content of 10.0 °Bx, inoculum ratio of *Lcb. paracasei* ZY to *Lcb. rhamnosus* SN12 as 1:1, inoculum size of 6 Log CFU/mL and fermentation time of 96 h. These results not only offer proper levels for each factor but also bring ideal guidance for RSM.

### 3.5. Optimization of GABA Content in Orange Juice Using RSM

#### 3.5.1. Analysis of Plackett–Burman Design

The results obtained by the Plackett–Burman design are shown in Table A4, and the multiple linear regression equations between each factor and the response values are as follows:

Y = 1.12 − 0.0586A − 0.0491B − 0.0618C + 0.0181D + 0.0137E − 0.0269F

As shown in Table 1, the analysis results of ANOVA suggest that the overall model is significant (*p* < 0.05). The coefficient of determination (R^2^) of the model is 0.9140, demonstrating a good match between the model and the data. The adjusted coefficient of determination (adjusted R^2^) is 0.8109, and this value implies that the model explains 81.09% of the data variance. The coefficient of variation (CV) is 4.41%, further proof of its repeatability and reliability [33]. The *p*-values of initial pH (A), fermentation temperature (B), and soluble solids content (C) are 0.0094, 0.0186, and 0.0076, respectively (*p* < 0.05), indicating that these three factors all have a significant effect on the response value. This result represents the fact that initial pH, fermentation temperature, and soluble solids content are the significant factors influencing GABA accumulation in fermented orange juice.

#### 3.5.2. Analysis of Box–Behnken Design

The results obtained by the Plackett–Burman design are shown in Table A6. A total of 17 runs for experimentation have been conducted to estimate the GABA content of fermented orange juice as response values (Y). The variables include initial pH (A), fermentation temperatures (B), and soluble solids contents (C). The following nonlinear regression equation was fitted:

Y = 0.7765 + 0.0240A + 0.0109B + 0.0085C − 0.0197AB + 0.0128AC − 0.0082BC − 0.0323A^2^ − 0.0276B^2^ − 0.0188C^2^

Through ANOVA analysis of the Box–Behnken design (Table 2), the model had an *F*-value of 9.85 and a *p*-value of 0.0032, which indicates that the model is extremely significant. The lack of fit was not significant (*p* > 0.05), suggesting that the model fits the data well and is reliable. The model’s R^2^ is 0.9268, and its adjusted R^2^ (0.8327) is close to this value, indicating the model is robust and can explain 83.27% of the variation in the response value. The predictive coefficient of determination (predictive R^2^) is 0.7656, further validating the model’s predictive performance. The CV value is 1.96%, which is below 5%, showing that the experimental data exhibit good precision and repeatability. It is well-suited for application in the process optimization of GABA production from orange juice. Significance analysis revealed that the initial pH (A), the quadratic term of initial pH (A^2^), and the quadratic term of fermentation temperature (B^2^) exert extremely significant effects on GABA production (*p* < 0.01), while the interaction between initial pH and fermentation temperature (AB) and the quadratic term of soluble solids content (C^2^) exert significant effects (*p* < 0.05). Based on the *F*-values of each factor for GABA production, the degree of influence of each factor on GABA production is as follows: initial pH > fermentation temperature > soluble solids content.

**Figure 4:**
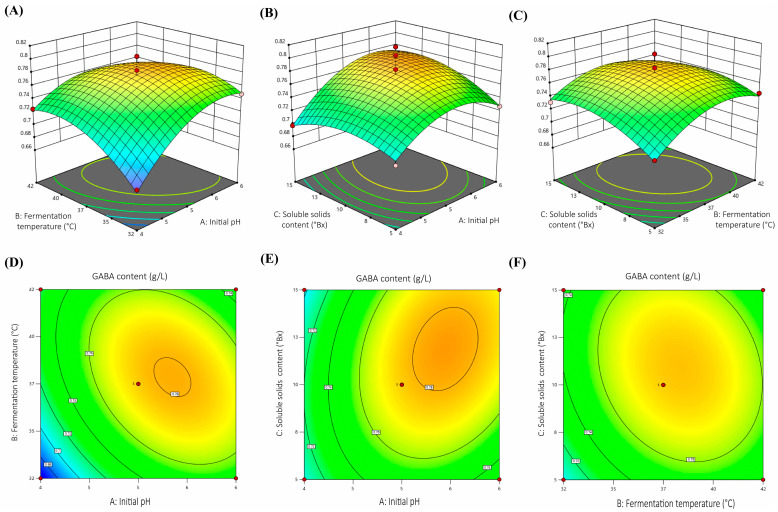
Response surface and contour plots showing interactive effects of initial pH and fermentation temperature (**A**,**D**), initial pH and soluble solids content (**B**,**E**), and fermentation temperature and soluble solids content (**C**,**F**) on GABA production. Red and pink dots in the response surface plots (**A**–**C**) denote observed values above and below the predicted surface, respectively; red dots in the contour plots (**D**–F) correspond to experimental design points. The color transition from blue to yellow indicates increasing GABA content.

To clarify the effects of significant factors’ interactions on GABA production, response surface and contour plots are presented (Figure 4). The steeper the slope on the response surface plot, the greater the effect of that factor on GABA content, thereby aiding in understanding the interactions among various factors [33]. Analysis results indicated that mutual interactive effects existed between each pair of factors. The interactive effect of initial pH and fermentation temperature is illustrated in Figure 4A,D, revealing that the GABA yield reached the highest level under weakly acidic conditions (pH 5.0–6.0) and within the temperature range of 30–38 °C. As shown in Figure 4B,E, the interactive effect of initial pH and soluble solids content demonstrated that the effect of soluble solids content on GABA yield gradually enhanced as the pH value approached neutrality. As presented in Figure 4C,F, the contour lines between fermentation temperature and soluble solids content tend to be circular, indicating a relatively weak interactive effect between these two factors. The analysis of response surface and contour plots indicates that fermentation temperature has the largest influence on GABA production, followed by initial pH, and finally soluble solids content. This order differs slightly from the ranking based on *F*-values because the response surface plots better reflect the actual strength of combined effects under real experimental conditions.

The optimal fermentation conditions, determined by combining single-factor experiments with RSM, were initial pH 5.46, fermentation temperature 36.89 °C, soluble solids content 11.92 °Bx, inoculum ratio of *Lcb. paracasei* ZY to *Lcb. rhamnosus* SN12 1:1, inoculum size 6 Log CFU/mL, and fermentation time 96 h. Under these conditions, the model estimated that the maximum GABA production would reach 0.78 g/L.

### 3.6. Effect of Optimal Fermentation Conditions on GABA Content in Orange Juice

**Figure 5:**
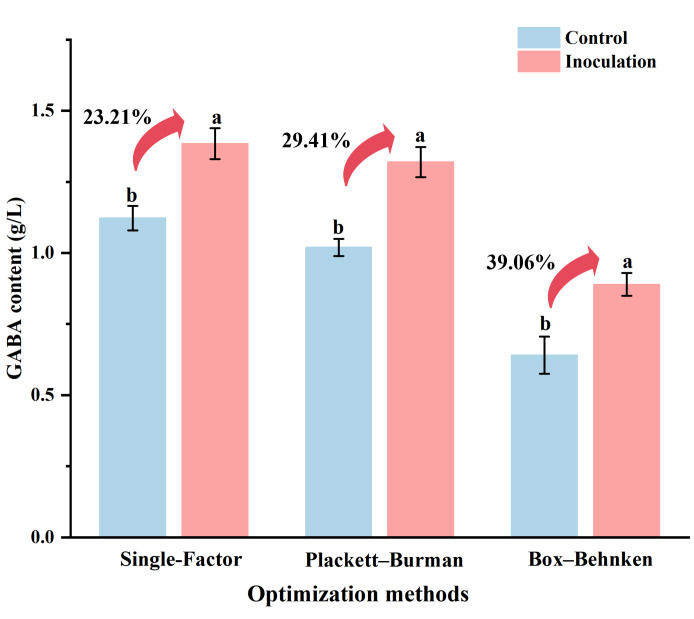
Enhancement of GABA content in fermented orange juice by different optimization methods. Different letters indicate significant differences before and after the same optimization methods (*p* < 0.05). “Control” represents orange juice without bacterial inoculation, while “inoculation” represents orange juice inoculated with LAB and subjected to optimized fermentation.

To assess the reliability and practical usefulness of the RSM, fermentation experiments were conducted under the optimized conditions. These included initial pH 5.5, fermentation temperature 37 °C, soluble solids content 12.0 °Bx, inoculum ratio of *Lcb. paracasei* ZY to *Lcb. rhamnosus* SN12 1:1, inoculum size 6 Log CFU/mL, and fermentation time 96 h. Uninoculated orange juice was used as the control. The results showed that the GABA content in the fermented orange juice reached 0.89 g/L, representing an increase of 39.06% compared with the uninoculated orange juice (0.65 g/L GABA) (Figure 5).

Different optimization methods had different effects on GABA production. As shown in Figure 5, the highest GABA content (1.38 g/L) was achieved at a soluble solids content of 10.0 °Bx in the single-factor experiments, representing a 23.21% increase compared to uninoculated orange juice (1.12 g/L). The Plackett–Burman design yielded a GABA content of 1.33 g/L, which corresponds to a 29.41% increase. Although the initial GABA content prior to Box–Behnken optimization was relatively low (0.65 g/L), this method achieved the most significant enhancement, with a 39.06% increase. Wang et al. [21] optimized the fermentation process of soymilk using *Lpb. plantarum* Lp 3, yielding a final GABA concentration of 0.55 g/L, which represents an approximately 20.00% improvement compared to the unfermented control. In another study, Jin et al. [19] fermented litchi juice with *L. brevis* LBJ-29 for 48 h and reported a 10-fold increase in GABA content relative to the unfermented counterpart. The high GABA yields observed in these studies may be attributed to the strains’ robust metabolic activity and efficient substrate utilization. However, since *L. brevis* is not generally recognized as a food-grade microorganism, its application in the food industry remains limited. While *Lpb. plantarum* Lp 3, *Lcb. paracasei* ZY, and *Lcb. rhamnosus* SN12 exhibit a lower GABA conversion capacity than *L. brevis* LBJ-29, process optimization remains a viable and effective strategy to enhance GABA yield. While single-factor optimization facilitates the rapid identification of reasonable ranges and levels for individual factors, RSM is more effective than the former in examining the interactions between various factors.

## 4. Conclusions

This study increased the content of GABA in fermented orange juice by optimizing the fermentation process. A total of fifty-two LAB strains were screened for GABA production using the Berthelot colorimetric method, and forty-three strains showed GABA-producing ability. The top fifteen strains were further analyzed by HPLC. *Lcb. paracasei* ZY and *Lcb. rhamnosus* SN12 exhibited high and stable GABA-producing ability and were selected for orange juice fermentation. Single-factor experiments were conducted to evaluate the effects of initial pH, fermentation temperature, soluble solids content, inoculum ratio, inoculum size, and fermentation time on GABA production in fermented orange juice. Through Plackett–Burman design analysis, initial pH, fermentation temperature, and soluble solids content were identified as the significant factors affecting GABA accumulation. The further optimization of these three factors was performed by Box–Behnken designs. The final optimized fermentation conditions are as follows: initial pH of 5.5, fermentation temperature of 37 °C, soluble solids content of 12.0 °Bx, inoculum ratio of *Lcb. paracasei* ZY to *Lcb. rhamnosus* SN12 as 1:1, inoculum size of 6 Log CFU/mL, and fermentation time of 96 h. Under these conditions, the GABA content in fermented orange juice increased to 0.89 g/L, which was 39.06% higher than that in uninoculated orange juice. This research not only screened out excellent strains for the development of functional juices, but also provided process references and technical support for the industrial production of functional products by enterprises.

## Figures and Tables

**Table 1:** Analysis of variance (ANOVA) for the Plackett–Burman design.

Source	Sum of Squares	Df	Mean Square	*F*-Value	*p*-Value	Significance
Model	0.1310	6	0.0218	8.86	0.0150	Significant
A-Initial pH	0.0413	1	0.0413	16.74	0.0094	**
B-Fermentation temperature	0.0290	1	0.0290	11.76	0.0186	*
C-Soluble solids content	0.0459	1	0.0459	18.62	0.0076	*
D-Starter ratio	0.0039	1	0.0039	1.60	0.2623	
E-Inoculum size	0.0023	1	0.0023	0.91	0.3830	
F-Fermentation time	0.0087	1	0.0087	3.54	0.1188	
Residual	0.0123	5	0.0025			
Cor Total	0.1433	11				
R^2^	0.9140					
Adjusted R^2^	0.8109					
Predicted R^2^	0.5049					
C.V.%	4.41					

Note: * indicates a significant difference at *p* < 0.05, and ** indicates a highly significant difference at *p* < 0.01.

**Table 2:** Analysis of variance (ANOVA) for the quadratic model of Box–Behnken design.

Source	Sum of Squares	Df	Mean Square	*F*-Value	*p*-Value	Significance
Model	0.0187	9	0.0021	9.85	0.0032	Significant
A-Initial pH	0.0046	1	0.0046	21.88	0.0023	**
B-Fermentation temperature	0.0009	1	0.0009	4.48	0.0721	
C-Soluble solids content	0.0006	1	0.0006	2.72	0.1433	
AB	0.0015	1	0.0015	7.33	0.0303	*
AC	0.0007	1	0.0007	3.09	0.1220	
BC	0.0003	1	0.0003	1.28	0.2959	
A^2^	0.0044	1	0.0044	20.82	0.0026	**
B^2^	0.0032	1	0.0032	15.23	0.0059	**
C^2^	0.0015	1	0.0015	7.07	0.0325	*
Residual	0.0015	7	0.0002			
Lack of fit	0.0002	3	0.0001	0.17	0.9108	Not significant
Pure error	0.0013	4	0.0003			
Cor toal	0.0202	16				
R^2^	0.9268					
Adjusted R^2^	0.8327					
Predicted R^2^	0.7656					
C.V.%	1.96					

Note: * indicates a significant difference at *p* < 0.05, and ** indicates a highly significant difference at *p* < 0.01.

## Data Availability

The original contributions presented in the study are included in the article, further inquiries can be directed to the corresponding authors.

## Appendix A

**Table A1:** Information on the strains used in this study.

Number	Strain Name	Isolation Source	Acquisition Method
1	*Lacticaseibacillus paracasei* ZY	Fermented milk	Laboratory isolation
2	*Lacticaseibacillus rhamnosus* SN12	Fermented milk	Laboratory isolation
3	*Lactiplantibacillus plantarum* M35	Fermented meat	Laboratory isolation
4	*Lactiplantibacillus plantarum* 2-22-LJ	Fermented meat	Laboratory isolation
5	*Lactiplantibacillus plantarum* Z5-24	Fermented meat	Laboratory isolation
6	*Lacticaseibacillus paracasei* 3-4	Fermented meat	Laboratory isolation
7	*Lactiplantibacillus plantarum* M7	Fermented meat	Laboratory isolation
8	*Lacticaseibacillus paracasei* 3-9	Fermented meat	Laboratory isolation
9	*Lactiplantibacillus plantarum* LS3-1	Fermented pickles	Laboratory isolation
10	*Lactiplantibacillus plantarum* 3-2-LJ	Fermented meat	Laboratory isolation
11	*Limosilactobacillus reuteri* PC1	Fermented pickles	Laboratory isolation
12	*Lactiplantibacillus plantarum* HJZ1	Distiller’s grains	Laboratory isolation
13	*Lacticaseibacillus paracasei* K22	Fermented milk	Laboratory isolation
14	*Lactiplantibacillus plantarum* Z5-25	Fermented meat	Laboratory isolation
15	*Lacticaseibacillus casei* K34	Fermented milk	Laboratory isolation
16	*Lacticaseibacillus paracasei* K38	Fermented milk	Laboratory isolation
17	*Pediococcus acidilactici* K2	Fermented meat	Laboratory isolation
18	*Lactiplantibacillus plantarum* 3-19-LJ	Fermented meat	Laboratory isolation
19	*Lacticaseibacillus paracasei* MAS-B2	Fermented milk	Laboratory isolation
20	*Pediococcus pentosaceus* 2-10-2-LJ	Fermented meat	Laboratory isolation
21	*Limosilactobacillus fermentum* CGMCC1.8	——	Obtained from CGMCC
22	*Lactiplantibacillus plantarum* J11	Fermented meat	Laboratory isolation
23	*Lacticaseibacillus rhamnosus* H7	Fermented meat	Laboratory isolation
24	*Lactococcus lactis subsp. lactis* MAS2-2	Fermented meat	Laboratory isolation
25	*Lactiplantibacillus plantarum* 1-26-LJ	Fermented meat	Laboratory isolation
26	*Lactiplantibacillus plantarum* N-M-1	Fermented milk	Laboratory isolation
27	*Latilactobacillus sakei* PC19	Fermented pickles	Laboratory isolation
28	*Lactiplantibacillus plantarum* PC14	Fermented pickles	Laboratory isolation
29	*Lactobacillus acidophilus* A20	Fermented meat	Laboratory isolation
30	*Lacticaseibacillus paracasei* Z3-11	Fermented meat	Laboratory isolation
31	*Lactiplantibacillus plantarum* 1-1	Fermented milk	Laboratory isolation
32	*Lacticaseibacillus rhamnosus* SN9	Fermented pickles	Laboratory isolation
33	*Streptococcus salivarius subsp.thermophilus* M17-11	Fermented milk	Laboratory isolation
34	*Lactiplantibacillus plantarum* 2-14-LJ	Fermented meat	Laboratory isolation
35	*Lactiplantibacillus plantarum* 1-11	Fermented milk	Laboratory isolation
36	*Pediococcus pentosaceus* HJZ2	Distiller’s grains	Laboratory isolation
37	*Lactiplantibacillus plantarum* M19	Fermented meat	Laboratory isolation
38	*Streptococcus salivarius subsp. thermophilus* KF17-2	——	Obtained from CICC
39	*Latilactobacillus sakei* S13	Fermented milk	Laboratory isolation
40	*Lacticaseibacillus paracasei* Z3-9	Fermented meat	Laboratory isolation
41	*Lacticaseibacillus paracasei* K23	Fermented milk	Laboratory isolation
42	*Lactobacillus delbrueckii subsp.bulgaricus* 6103	——	Obtained from CICC
43	*Lacticaseibacillus rhamnosus* SN13	Fermented pickles	Laboratory isolation
44	*Lactobacillus helveticus* 6102	——	Obtained from CICC
45	*Lacticaseibacillus paracasei* Z3-4	Fermented meat	Laboratory isolation
46	*Limosilactobacillus fermentum* 2-34-LJ	Fermented meat	Laboratory isolation
47	*Lactobacillus acidophilus* LS2-3	Fermented pickles	Laboratory isolation
48	*Lactiplantibacillus plantarum* A72	Fermented pickles	Laboratory isolation
49	*Latilactobacillus sakei* PC2	Fermented pickles	Laboratory isolation
50	*Latilactobacillus sakei* PC3	Fermented pickles	Laboratory isolation
51	*Latilactobacillus sakei* PC17	Fermented pickles	Laboratory isolation
52	*Latilactobacillus sakei* PC30	Fermented pickles	Laboratory isolation

Note: CGMCC refers to the China General Microbiological Culture Collection Center; CICC refers to the China Industrial Microbial Culture Collection Center.

**Table A2:** Experimental factors and levels in the single-factor design.

Factors	Unit	Levels				
Initial pH	—	3.0	4.0	5.0	6.0	7.0
Fermentation temperature	°C	27	32	37	42	47
Soluble solids content	°Bx	5.0	10.0	15.0	20.0	25.0
Inoculum ratio	—	1:0	1:1	1:2	2:1	0:1
Inoculum size	Log CFU/mL	3	6	9	12	15
Fermentation time	h	48	72	96	108	120

**Table A3:** Experimental factors and coded levels in the Plackett–Burman design.

Symbols	Factors	Unit	Coded Levels	
A	Initial pH	—	5.0	7.0
B	Fermentation temperature	°C	37	47
C	Soluble solids content	°Bx	10.0	25.0
D	Inoculum ratio	—	1:0	1:1
E	Inoculum size	Log CFU/mL	6	15
F	Fermentation time	h	96	120

**Table A4:** Plackett–Burman design and experimental results.

Run	A	B	C	D	E	F	GABA Content (g/L)
1	1	−1	−1	−1	1	−1	1.18 ± 0.02
2	−1	1	−1	1	1	1	1.2 ± 0.05
3	−1	1	1	−1	1	−1	1.07 ± 0.10
4	1	−1	1	1	1	−1	1.14 ± 0.06
5	1	1	−1	−1	−1	1	1.06 ± 0.04
6	−1	−1	−1	1	1	1	1.29 ± 0.06
7	−1	−1	−1	−1	−1	−1	1.32 ± 0.10
8	−1	−1	1	−1	−1	1	1.06 ± 0.01
9	1	1	1	−1	1	1	0.92 ± 0.07
10	−1	1	1	1	−1	−1	1.13 ± 0.08
11	1	−1	1	1	−1	1	1.03 ± 0.10
12	1	1	−1	1	−1	−1	1.04 ± 0.14

**Table A5:** Experimental factors and coded levels in the Box–Behnken design.

Symbols	Factors	Unit	Coded Levels		
			−1	0	1
A	Initial pH	—	4.0	5.0	6.0
B	Fermentation temperature	°C	32	37	42
C	Soluble solids content	°Bx	5.0	10.0	15.0

**Table A6:** Box–Behnken design and experimental results.

Run	Initial pH	Fermentation Temperature (°C)	Soluble Solids Content (°Bx)	GABA Content (g/L)
1	−1	−1	0	0.67 ± 0.04
2	1	0	1	0.78 ± 0.12
3	−1	1	0	0.72 ± 0.08
4	0	0	0	0.80 ± 0.08
5	1	0	−1	0.73 ± 0.03
6	0	1	1	0.74 ± 0.05
7	1	1	0	0.73 ± 0.10
8	0	1	−1	0.75 ± 0.04
9	0	0	0	0.77 ± 0.05
10	0	0	0	0.76 ± 0.07
11	−1	0	1	0.70 ± 0.11
12	0	−1	−1	0.70 ± 0.13
13	0	0	0	0.78 ± 0.07
14	0	−1	1	0.73 ± 0.09
15	−1	0	−1	0.70 ± 0.06
16	0	0	0	0.77 ± 0.10
17	1	−1	0	0.75 ± 0.07
